# Angiopoietin-like protein 2 regulates *Porphyromonas gingivalis* lipopolysaccharide-induced inflammatory response in human gingival epithelial cells

**DOI:** 10.1371/journal.pone.0184825

**Published:** 2017-09-21

**Authors:** Tasuku Ohno, Genta Yamamoto, Jun-ichiro Hayashi, Eisaku Nishida, Hisashi Goto, Yasuyuki Sasaki, Takeshi Kikuchi, Mitsuo Fukuda, Yoshiaki Hasegawa, Makio Mogi, Akio Mitani

**Affiliations:** 1 Department of Periodontology, School of Dentistry, Aichi Gakuin University, Chikusa-ku, Nagoya, Aichi, Japan; 2 Department of Microbiology, School of Dentistry, Aichi Gakuin University, Nagoya, Chikusa-ku, Aichi, Japan; 3 Department of Integrative Education of Pharmacy, School of Pharmacy, Aichi Gakuin University, Chikusa-ku, Nagoya, Aichi, Japan; Universita degli Studi di Napoli Federico II, ITALY

## Abstract

Angiopoietin-like protein 2 (ANGPTL2) maintains tissue homeostasis by inducing inflammation and angiogenesis. It is produced in infiltrating immune cells or resident cells, such as adipocytes, vascular endothelial cells, and tumor cells. We hypothesized that ANGPTL2 might play an important role as a unique mediator in both systemic and periodontal disease. We demonstrated an increased ANGPTL2 concentration in gingival crevicular fluid from chronic periodontitis patients. *Porphyromonas gingivalis* lipopolysaccharide (LPS) treatment strongly induced ANGPTL2 mRNA and protein levels in Ca9-22 human gingival epithelial cells. Recombinant human ANGPTL2 increased interleukin 1β (IL-1β), IL-8, and tumor necrosis factor-α (TNF-α) mRNA and protein levels in Ca9-22 cells. Small-interfering (si)RNA-mediated ANGPTL2 knockdown in Ca9-22 cells reduced IL-1β, IL-8 and TNF-α mRNA and protein levels compared with control siRNA (*p*<0.01) in *P*. *gingivalis* LPS-stimulated Ca9-22 cells. Antibodies against integrin α5β1, an ANGPTL receptor, blocked induction of these inflammatory cytokines in *P*. *gingivalis* LPS-treated Ca9-22 cells, suggesting that secreted ANGPTL induces inflammatory cytokines in gingival epithelial cells via an autocrine loop. The classic sequential cascade of *P*. *gingivalis* LPS → inflammatory cytokine induction is well established. However, in the current study, we reveal a novel cascade comprising sequential *P*. *gingivalis* LPS → ANGPTL2 → integrin α5β1 → inflammatory cytokine induction, which might be responsible for inducing potent periodontal disorganization activity in gingival epithelial cells. Via this pathway, ANGPTL2 functions in the pathogenesis of periodontitis and contributes to prolonging chronic inflammation in patients with systemic disease.

## Introduction

Periodontitis is a chronic inflammatory disease caused by periodontal pathogens. In this disease, the inflammatory response is the major contributor to structural component damage in the periodontium [1]. Gingival epithelial cells are the initial barriers to oral microbial intrusion, and their modulatory function in the inflammatory response in periodontal diseases has recently been highlighted [2]. *Porphyromonas gingivalis*, a gram-negative anaerobic bacterium, has been identified as one of the most important periodontal pathogens. *P*. *gingivalis* induces the release of large numbers of outer membrane vesicles containing lipopolysaccharide (LPS), which can penetrate periodontal tissue [3, 4]. *P*. *gingivalis* LPS is reported to induce an inflammatory response in various cell types (such as macrophages, fibroblasts, endothelial cells, and gingival epithelial cells) via activating Toll-like receptor 2 (TLR2) or TLR4. These combined inflammatory responses produce imbalance between osteoblast and osteoclast numbers, resulting in alveolar bone resorption [5]. Periodontal disease is influenced by both systemic and environmental factors such as age, smoking, diabetes mellitus, and overweight or obesity [6]. Clinical studies have revealed an association between periodontal disease and systemic diseases such as diabetes, cardiovascular disease, metabolic syndrome, and cancer [7–13]. There is also increasing evidence that periodontal disease is an independent risk-factor for several systemic diseases, which is changing our understanding about the causality and directionality of associated oral and systemic diseases [14].

The angiopoietin-like protein (ANGPTL) family of eight secreted glycoproteins was recently identified [15, 16]. ANGPTLs do not bind to the Tie2 angiopoietin receptor or to the related protein Tie1, and are classified as orphan ligands; thus, they appear to have distinct biological functions from angiopoietins [17]. ANGPTL2 maintains tissue homeostasis by inducing inflammation and angiogenesis [17, 18]. In some contexts, ANGPTL2 overexpression promotes irreversible pathological tissue remodeling via integrin α5β1 and the development or progression of metabolic diseases, type 2 diabetes, atherosclerotic vascular disease, and some cancers caused by chronic inflammation [16, 19–22]. ANGPTL2 expression in increased in infiltrating immune cells or resident cells, such as adipocytes, vascular endothelial cells, and tumor cells [19, 21, 22]. It is therefore likely that enhanced ANGPTL2 autocrine or paracrine signaling accelerates disease development and progression. However, no studies have yet investigated a role for ANGPTL2 in the pathogenesis of inflammatory disease, including periodontal disease. Thus, further studies are required to understand its intracellular function.

We tested the hypothesis that ANGPTL2 is a unique mediator in periodontal disease by conducting a series of in vivo and in vitro experiments. We found increased ANGPTL2 concentrations in gingival crevicular fluid (GCF) samples from chronic periodontitis (CP) patients.

## Materials and methods

### Participants

CP patients (at least six teeth containing sites with a probing depth [PD] of ≥5 mm, a clinical attachment level [CAL] of ≥6 mm, and extensive bone loss by radiography) and healthy adult volunteers were recruited from the outpatient clinic of Aichi Gakuin University Dental Hospital, Nagoya, Japan. Exclusion criteria were: (1) smoking within the past 5 years; (2) antibiotic therapy in the last 6 months; (3) pregnancy; and (4) any systemic condition that could affect periodontitis progression (e.g. immunologic disorders, diabetes, or osteoporosis). Healthy volunteers had no signs of clinical periodontal attachment loss, a PD of ≤3 mm, percentage of bleeding on probing (full mouth) of <10%, and maximal bone loss (full mouth) of <10%. A single examiner recorded PD, CAL, and demographic data for all participants. A total of 46 participants comprising 24 CP patients (14 men and 10 women, mean age 55.4 ± 14.4 years) and 22 healthy volunteers (13 men and 9 women, mean age 44.2 ± 18.5 years) were included in the study.

Informed written consent was obtained from all participants at the first outpatient visit, and the study protocol for human participants was reviewed and approved by Aichi Gakuin University, School of Dentistry, Ethics Committee (permit number AGUD 383).

### GCF sampling

GCF samples were collected from three buccal sites (mesial, center, and distal) of teeth with a single root, a PD of ≤3 mm (for all three sites; healthy individuals) or ≥4 mm (at least one site; CP patients), and prominent radiographic evidence of alveolar bone loss (CP patients only), based on a previously described method [23]. GCF samples were collected from three sites identified in the clinical examination by inserting paper strips (Oraflow, Hewlett, NY, USA) into the gingival crevice until mild resistance was felt and removed after 30 s. The volumes of GCF samples were quantified using a Periotron 8000 (Oraflow) that had been calibrated with pooled human serum. ANGPTL2 protein levels in these samples were measured (described below).

### Cell culture and treatment

The Ca9-22 human oral epithelial cell line was purchased from the RIKEN BRC Cell Bank (Tsukuba, Japan). Ca9-22 cells were maintained in Dulbecco’s modified Eagle’s medium (GibcoBRL, Grand Island, NY, USA) containing 10% fetal bovine serum (Hyclone Laboratories, Logan, UT, USA), penicillin (100 U/ml), and streptomycin (100 μg/ml) in a humidified atmosphere containing 5% CO_2_ at 37°C, which was replaced every 3 days. Human primary gingival epithelial cells (HGECs; CELLnTEC, Bern, Switzerland) were maintained in CnT-Prime epithelial culture medium (CELLnTEC), which was replaced every 5 days, in a humidified atmosphere containing 5% CO_2_ at 37°C. For experiments, cells were seeded into 12-well plates at 1×10^5^ cells/ml, incubated for 48 h, primed with 50 ng/ml recombinant human (rh) interferon (IFN) γ (PeproTech Rocky Hill, NJ, USA) for 12 h, and then treated with *P*. *gingivalis* 1690 (penta-acylated lipid A) LPS (0–1 μg/ml; Astarte Biologics, Bothell, WA, USA). It is conceivable that gingival epithelial cells in inflamed gingival tissues are already primed (with IFN-γ produced by inflammatory lymphoid cells) for secretion of various inflammatory cytokines in response to bacterial components such as LPS [24, 25]. Therefore, we pretreated cells with rhIFN-γ [26]. We confirmed that IFN-γ treatment does not affect ANGPTL2 induction (data not shown).

### Quantitative polymerase chain reaction analysis

Quantitative polymerase chain reaction (qPCR) analysis was performed as previously described [27]. qPCR analyses using TaqMan Universal PCR master mix and TaqMan gene expression assays (Applied Biosystems, Foster City, CA, USA) were performed to quantify mRNA levels of human *ANGPTL2* (Hs00171912_m1), *IL1B* (Hs01555410_m1), *IL8* (Hs00174103_m1), *TNF* (Hs00174128_m1). All mRNA levels were normalized to 18S rRNA levels (Hs99999901_s1). Total RNA was isolated from Ca9-22 cells using the NucleoSpin RNA II system (Macherey-Nagel, Dueren, Germany) according to the manufacturer’s instructions. RNA quality was evaluated by measuring the A260/A280nm ratio using a fluorospectrometer (NanoDrop ND-1000; Thermo Scientific, Wilmington, DE, USA). cDNA was synthesized using 13.2 μl total RNA (40–60 ng/μl), 6 μl 5× First Strand buffer, 3 μl DTT (0.1 mM), 6 μl dNTP Mix (2.5 mM), 0.75 μl random primers, 1 μl SuperScript III Reverse Transcriptase (200 units; Invitrogen, Carlsbad, CA, USA), and 0.5 μl ribonuclease inhibitor (Invitrogen). The reaction was incubated at 37°C for 60 min followed by 5 min at 95°C. Quantitative PCR (qPCR) was performed using a reaction volume of 50 μl containing 2.5 μg cDNA, 28.2 μl TaqMan Universal Master Mix (Applied Biosystems, Foster City, CA, USA), and 2.2 μl each of TaqMan Gene Expression assay buffer (Applied Biosystems) in MicroAmp Optical 96-well reaction plates covered with MicroAmp optical adhesive film (Applied Biosystems) on an ABI Prism 7000 Sequence Detection System with version 1.0 software (Applied Biosystems). Cycle parameters were: 10 min at 95°C, followed by 40 cycles of 15 s at 95°C and 1 min at 60°C. Quantification was performed using the ΔΔC_q_ method, as previously described [28]. Briefly, the fold change in mRNA expression levels was determined as follows: 2^−ΔΔCq^, where ΔΔC_q_ = [(C_q_ target − C_q_ 18S rRNA (treated group)) − (C_q_ target − C_q_ 18S rRNA (control group))], where “C_q_” denotes the quantification cycle).

### Western blotting analysis

Western blotting was performed as previously described [27]. Ca9-22 cells were lysed in CelLytic M lysis buffer (Sigma-Aldrich, St. Louis, MO, USA) containing a protease inhibitor cocktail (Nacalai Tesque, Kyoto, Japan) and phosphatase inhibitor cocktail (Nacalai Tesque). Proteins were separated by sodium dodecyl sulfate polyacrylamide gel electrophoresis (Bio-Rad Laboratories, Hercules, CA, USA), transferred to polyvinylidene fluoride membranes (Bio-Rad Laboratories), and probed with rabbit anti-human ANGPTL2 polyclonal antibody (Proteintech Group, Chicago, IL, USA, Cat:12316-1-AP) or rabbit anti-human β-actin monoclonal antibody (Cell Signaling Technologies, Beverly, MA, USA, Cat:#7074S). β-actin (Cell Signaling Technologies, Cat:#3700S) was the loading control. Goat anti-rabbit IgG (Cell Signaling Technologies, Cat:#7076S) and goat anti-mouse IgG were used to detect ANGPTL2 and β-actin, respectively.

### Flow cytometry

Ca9-22 cells (100 μL, containing 5×10^5^ cells) were incubated with anti-CD282 phycoerythrin (Miltenyi Biotec, Bergisch Gladbach, Germany, Cat:130-099-017), anti-CD284 allophycocyanin (Miltenyi Biotec, Cat:130-100-150), anti-CD49e phycoerythrin (Miltenyi Biotec, Cat:130-092-215), anti-CD85d phycoerythrin (Miltenyi Biotec, Cat:130-100-567) or isotype control antibody (Miltenyi Biotec, Cat:130-097-225) and analysed by flow cytometry using a MACSQuant analyzer and MACSQuantify software version 2.4 (Miltenyi Biotec).

### Recombinant human ANGPTL2 treatment

At 48 h after plating, Ca9-22 cells were incubated with 10 ng/ml rhANGPTL2 (ProSpec, Ness-Ziona, Israel) for 1, 3, 6, 9, or 12 h. IL-1β, IL-8, and TNF-α mRNA and protein levels were measured in cell lysates and cell culture supernatants, respectively.

### Pretreatment with anti-human integrin α5β1 monoclonal antibody

Before stimulation with *P*. *gingivalis* LPS or rhANGPTL2, Ca9-22 cells were incubated for 1 h with 1 ng/ml anti-human integrin α5β1 monoclonal antibody (Millipore, Darmstadt, Germany, Cat: MAB1969). We confirmed that 1 ng/ml antibody was sufficient to block integrin α5β1 (data not shown).

### Small-interfering RNA assays

At 24 h after seeding into six-well plates, Ca9-22 cells were transfected with negative control or Silencer Select Predesigned small-interfering (si)RNA targeting TLR2, TLR4, or ANGPTL2 (Life Technology, Tokyo, Japan) using Lipofectamine 3000 (Life Technology), according to the manufacturer’s instructions. After incubation for 48 h, cells were primed with rhIFN-γ for 12 h, washed three times with PBS, and treated with 30 ng/ml *P*. *gingivalis* LPS for 24 h.

### Enzyme-linked immunosorbent assay

ANGPTL2 concentration in GCF and culture supernatant samples was determined using an ANGPTL2 enzyme-linked immunosorbent assay (ELISA) kit (Immuno-Biological Laboratories, Gunma, Japan). After stimulation with *P*. *gingivalis* LPS or rhANGPTL2, the inflammatory cytokine (IL-1β, IL-8, TNF-α) concentration in culture supernatant was measured using an ELISA kit (R&D Systems, Minneapolis, MN, USA).

### Statistical analysis

Statistical analyses were performed using SPSS for Windows, version 15.0. (Chicago, IL, USA). Differences among groups were examined by one-factor analysis of variance (ANOVA); Student’s *t*-test was used for comparisons. Data are expressed as means ± standard deviation (SD). Statistical significance was set at *p*<0.05.

## Results

### ANGPTL2 levels are increased in GCF from CP patients

**Fig 1:**
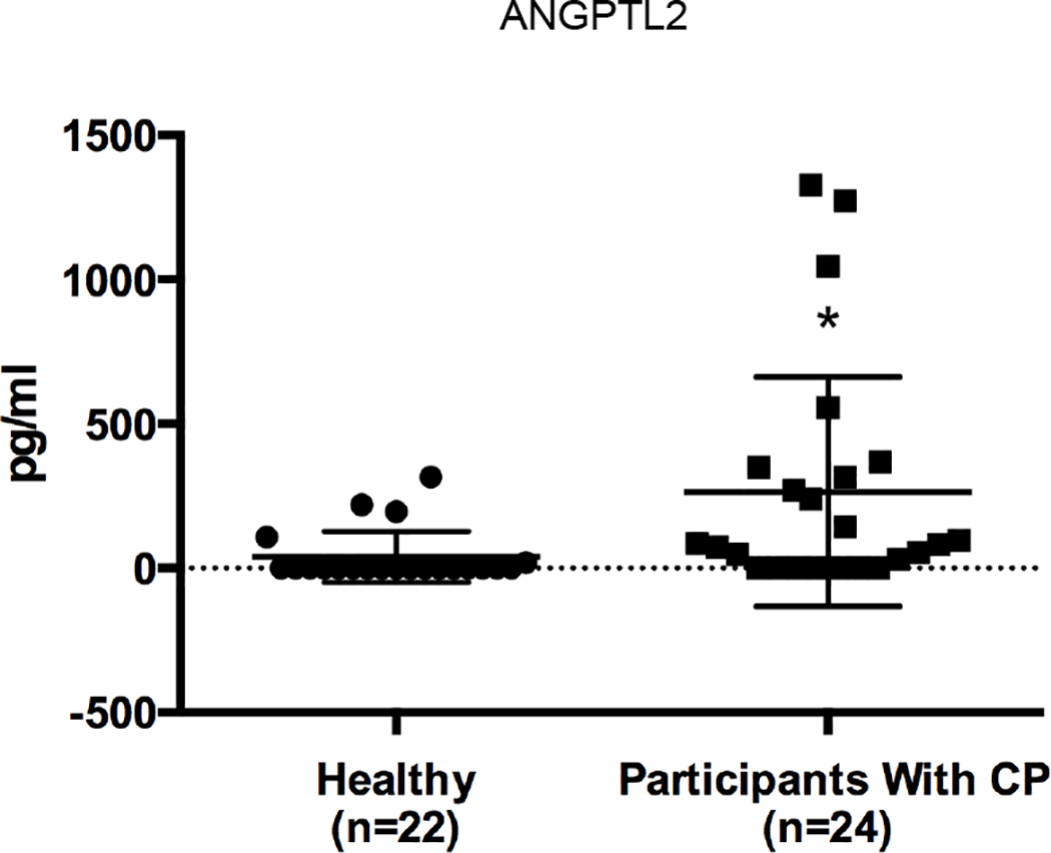
ANGPTL2 protein levels are increased in GCF from CP patients. ANGPTL2 protein levels were compared in GCF from CP patients and healthy individuals using the Student’s *t*-test. Data are expressed as means ± SD. *p < 0.05 vs control.

To examine whether ANGPTL2 protein levels are altered in CP patients, ANGPTL2 protein production was measured in GCF samples from 22 healthy participants and 24 CP patients. ANGPTL2 levels in healthy participants were relatively low (below the limit of detection in all but four), but were significantly higher in CP patients (*p*<0.05; Fig 1).

### *P*. *gingivalis* LPS stimulates ANGPTL2 in Ca9-22 cells and HGECs

**Fig 2:**
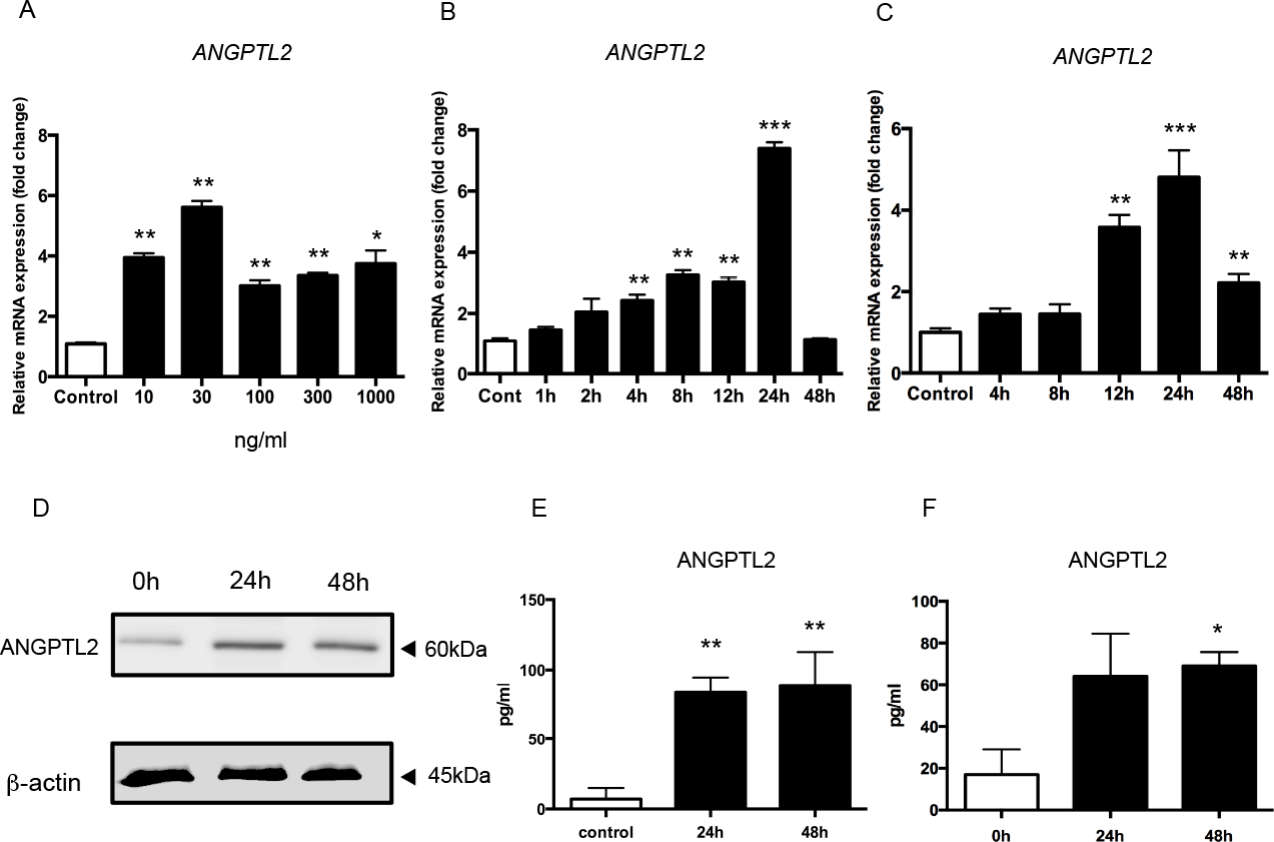
ANGPTL2 is upregulated by *P*. *gingivalis* LPS in Ca9-22 cells and HGECs. ANGPTL2 is upregulated by *P*. *gingivalis* LPS in Ca9-22 cells and HGECs. (A, B) *ANGPTL2* mRNA expression was measured in primed Ca9-22 cells stimulated with (A) 0, 10, 30, 100, 300, or 1000 ng/ml *P*. *gingivalis* LPS for 24 h or (B) 30 ng/ml *P*. *gingivalis* LPS for 0–48 h. (C) *ANGPTL2* mRNA expression was measured in primed HGECs stimulated with 30 ng/ml *P*. *gingivalis* LPS for 0–48 h. Differences among groups were analyzed using one-way ANOVA. Data are expressed as means ± SD (*n* = 3). *p < 0.05, **p < 0.01, *** *p* < 0.001 vs. control. (D) Western blot showing ANGPTL2 (60 kDa) levels after stimulation with 30 ng/ml *P*. *gingivalis* LPS for 24 and 48 h. (E) ANGPTL2 protein levels in Ca9-22 cells culture supernatant after 24 and 48 h stimulation with 30 ng/ml *P*. *gingivalis* LPS. (F) ANGPTL2 protein levels in HGEC culture supernatants at 24 and 48 h after stimulation with 30 ng/ml *P*. *gingivalis* LPS. Differences among groups were analyzed using one-way ANOVA. Data are expressed as means ± SD (n = 4). *p < 0.05, **p < 0.01 vs. control.

To investigate the relationship between altered ANGPTL2 mRNA and protein levels and CP, further in vitro experiments were performed using the Ca9-22 gingival epithelial cell line. *ANGPTL2* mRNA expression was measured in primed Ca9-22 cells stimulated with 0 (control), 10, 30, 100, 300, or 1000 ng/ml *P*. *gingivalis* LPS for 24 h. *ANGPTL2* mRNA induction was maximal in cells treated with 30 ng/ml (Fig 2A). At 4, 8, 12, and 24 h after treatment with 30 ng/ml *P*. *gingivalis* LPS, *ANGPTL2* mRNA levels were significantly higher in primed Ca9-22 cells than in control cells (Fig 2B). The *ANGPTL2* mRNA level was also significantly higher in primed HGECs than in control cells (Fig 2C).

Western blot analysis showed that ANGPTL2 (60 kDa) protein expression was also higher in primed Ca9-22 cells stimulated with *P*. *gingivalis* LPS compared with unstimulated controls (Fig 2D), indicating that *P*. *gingivalis* LPS induces ANGPTL2 protein. In addition, ANGPTL2 protein levels in culture supernatants from Ca9-22 cells and HGECs were increased at 24 and 48 h after stimulation with 30 ng/ml *P*. *gingivalis* LPS (Fig 2E and 2F). Because a similar trend was observed in the response of Ca9-22 cells and HGECs to *P*. *gingivalis* LPS stimulation, we mainly used Ca9-22 cells in the subsequent experiments.

### TLR2 and TLR4 siRNA reduce ANGPTL2 levels in Ca9-22 cells

**Fig 3:**
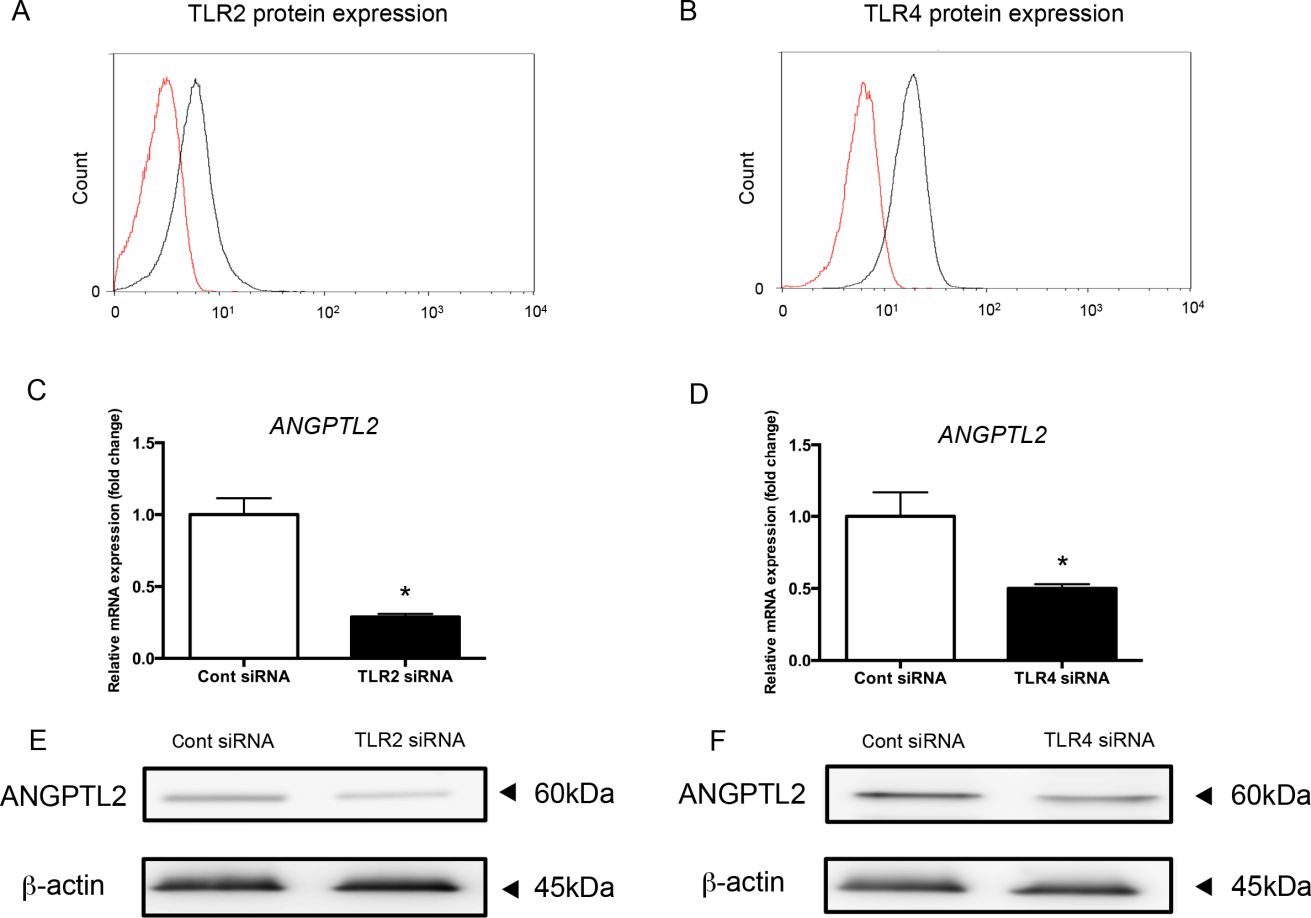
ANGPTL2 expression is suppressed by TLR2 or TLR4 siRNA in Ca9-22 cells. (A, B) TLR2 (A) and TLR4 (B) protein levels Ca9-22 cells transfected with TLR2 and TLR4 siRNA (red line), respectively, and control siRNA-transfected cells (black line). (C, D) *ANGPTL2* mRNA levels in Ca9-22 cells transfected with TLR2 and TLR4 siRNA after stimulation with 30 ng/ml *P*. *gingivalis* LPS for 24 h. Differences among groups were analyzed with the Student’s *t*-test. Data are expressed as means ± SD (*n* = 3). *p < 0.05 vs control. (E, F) Western blot analysis of ANGPTL2 protein levels in Ca9-22 cells transfected with TLR2 and TLR4 siRNA after stimulation with 30 ng/ml *P*. *gingivalis* LPS for 24 h.

Since *P*. *gingivalis* LPS is a TLR2 and TLR4 agonist, we knocked down TLR2 and TLR4 with siRNA in Ca9-22 cells. TLR2 and TLR4 expression was completely suppressed after siRNA treatment (Fig 3A and 3B). Both TLR2 and TLR4 siRNA strongly reduced *ANGPTL2* mRNA levels compared with control siRNA (Fig 3C and 3D). Western blot analysis confirmed that ANGPTL2 (60 kDa) protein levels were also decreased by TLR2 and TLR4 siRNA compared with control siRNA (Fig 3E and 3F). These data indicate that *P*. *gingivalis* LPS induction of ANGPTL2 is mediated by TLR2 and TLR4.

### rhANGPTL2 upregulates inflammatory cytokines in Ca9-22 cells

**Fig 4:**
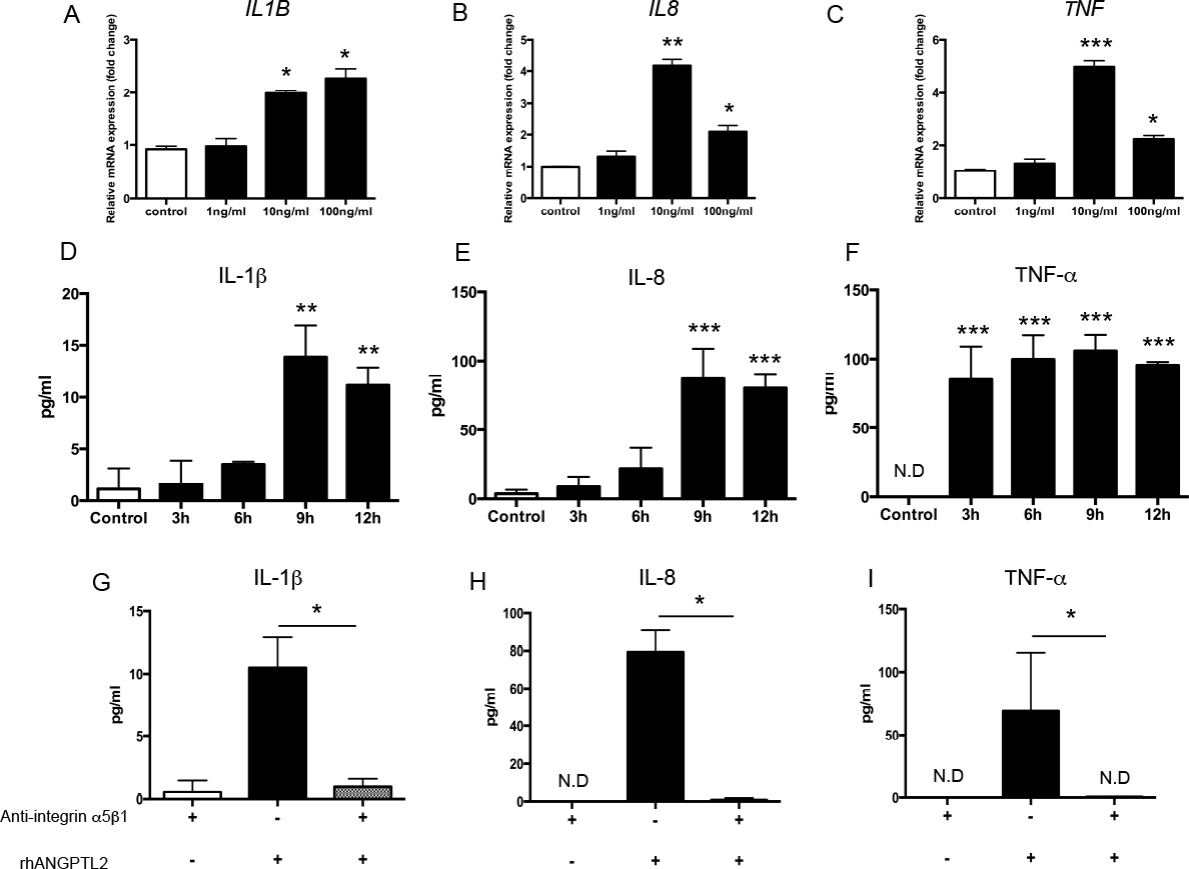
Inflammatory cytokines are upregulated by rhANGPTL2 in Ca9-22 cells. (A–C) mRNA levels of inflammatory cytokines were measured in Ca9-22 cells after stimulation with 0–100 ng/ml rhANGPTL2 for 1 h. (D–F) Protein levels of inflammatory cytokines were measured in Ca9-22 cells after stimulation with 10 ng/ml rhANGPTL2 for 3,6,9 and 12 h. Differences among groups were analyzed by one-way ANOVA. Data are expressed as means ± SD (*n* = 3). *p < 0.05, **p < 0.01, ***p < 0.001 vs. control. (G–I) Protein levels of inflammatory cytokines after anti-integrin α5β1 IgG pretreatment followed by stimulation with 10 ng/ml rhANGPTL2 for 9 h. Data are expressed as means ± SD (*n* = 4). *p < 0.05, **p < 0.01 vs. control. ND: not detectable.

To investigate the effect of ANGPTL2 on inflammatory cytokine secretion, Ca9-22 gingival epithelial cells were stimulated with 0–100 ng/ml rhANGPTL2 for 1 h. *IL1B*, *IL8*, and *TNF* mRNA levels were increased by rhANGPTL2 stimulation (Fig 4A–4C). IL-1β, IL-8 and TNF-α protein levels were also increased (Fig 4D–4F), indicating that ANGPTL2 increases inflammatory cytokine secretion.

We next confirmed that integrin α5β1 (the ANGPTL2 receptor) is expressed in Ca9-22 cells (data not shown). Pretreatment with anti-integrin α5β1 IgG completely suppressed the induction of inflammatory cytokines IL-1β, IL-8 and TNF-α in *P*. *gingivalis* LPS-stimulated Ca9-22 cells, confirming that interaction between ANGPTL2 and integrin α5β1 is needed to stimulate inflammatory cytokine production (Fig 4G–4I).

### ANGPTL2 siRNA downregulates inflammatory cytokines in Ca9-22 cells

**Fig 5:**
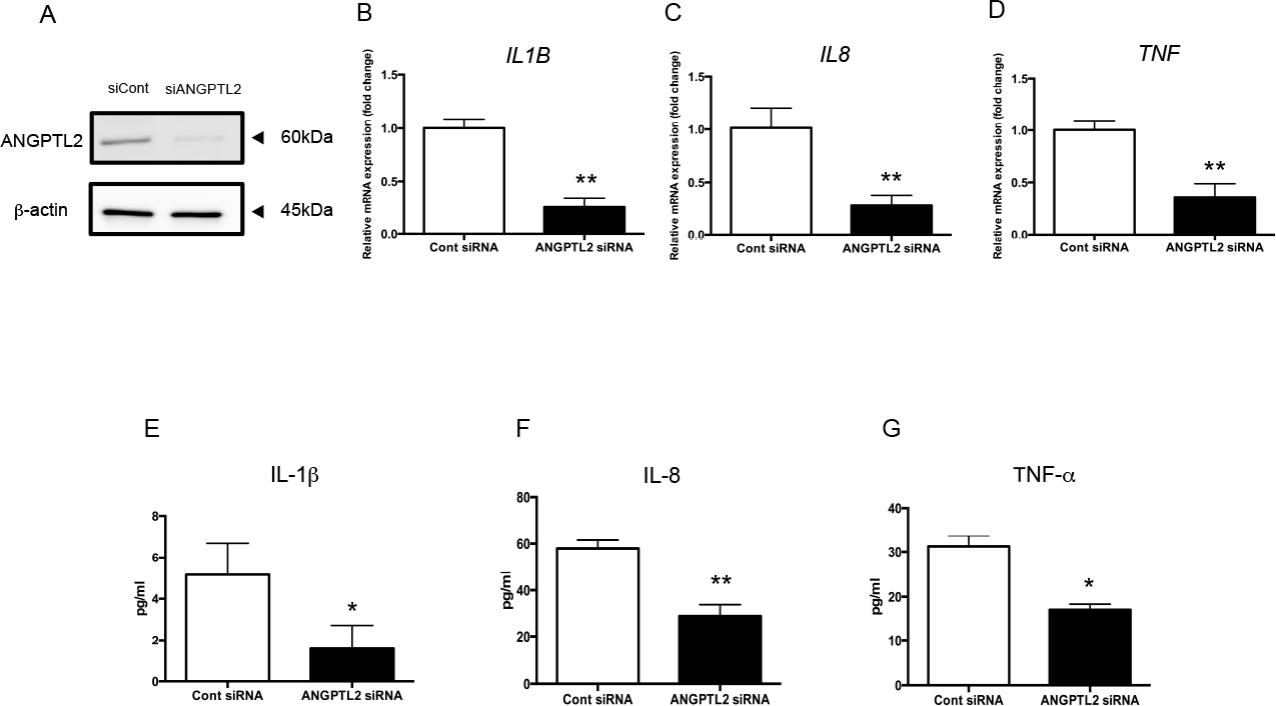
ANGPTL2 siRNA downregulates the inflammatory cytokine response to *P*. *gingivalis* LPS. (A) ANGPTL2 protein levels in Ca9-22 cells transfected with ANGPTL2 or control siRNA. (B–D) Inflammatory cytokine mRNA levels in Ca9-22 cells transfected with ANGPTL2 or control siRNA after stimulation with *P*. *gingivalis* LPS for 24 h. (E–G) Inflammatory cytokine protein levels in Ca9-22 cells transfected with ANGPTL2 or control siRNA after stimulation with *P*. *gingivalis* LPS for 24 h. Differences among groups were analyzed using the Student’s *t*-test. Data are expressed as means ± SD (*n* = 4). *p < 0.05, **p < 0.01 vs. control siRNA.

To examine whether *P*. *gingivalis* LPS-induced ANGPTL2 plays an important role in inflammatory cytokine production, ANGPTL2 was knocked down with siRNA in Ca9-22 cells. We next confirmed that ANGPTL2 protein expression is completely suppressed in cells treated with ANGPTL2 siRNA (Fig 5A). *IL1B*, *IL8*, and *TNF* mRNA levels were reduced in ANGPTL2 siRNA-treated Ca9-22 cells after *P*. *gingivalis* LPS stimulation compared with control siRNA-treated cells (*p*<0.01; Fig 5B–5D). IL-1β, IL-8, and TNF-α protein levels were also decreased ANGPTL2 siRNA-treated Ca9-22 cells compared with control siRNA-treated cells (*p*<0.05; Fig 5E–5G).

**Fig 6:**
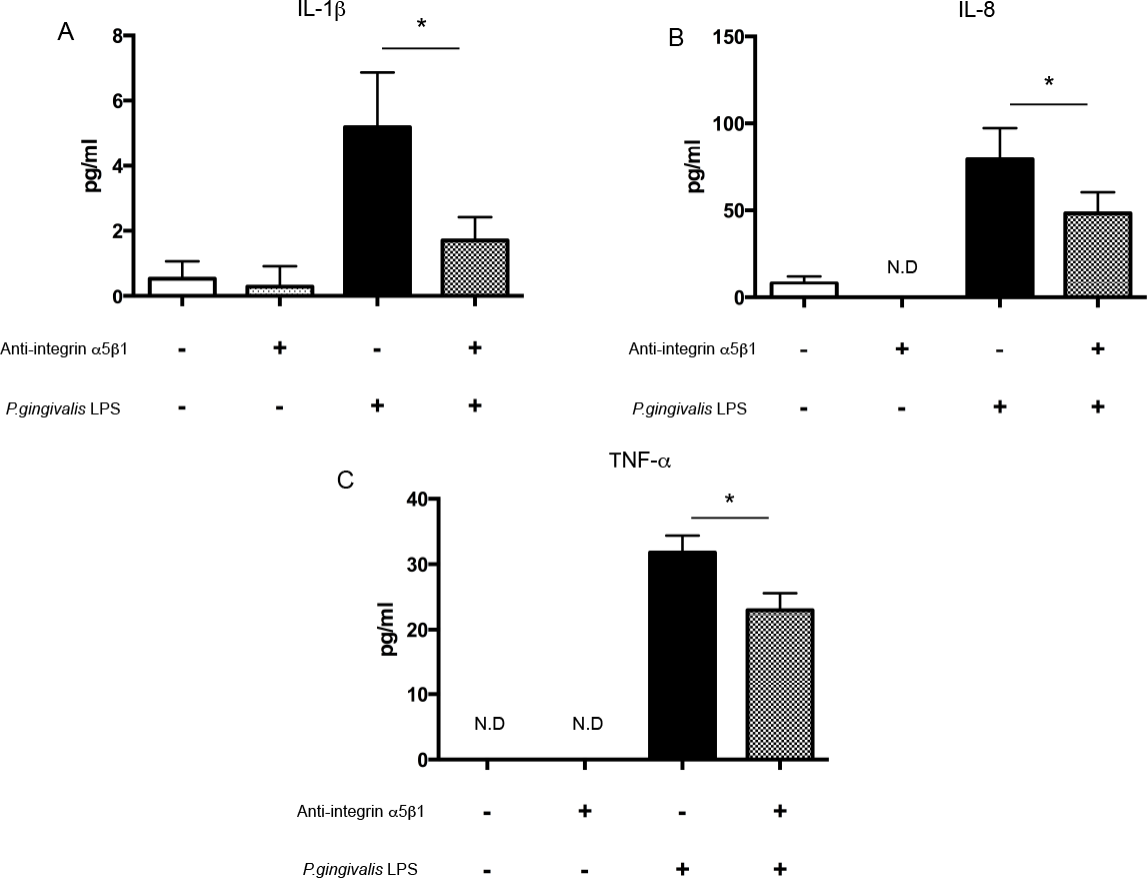
Inflammatory cytokines are downregulated by blocking integrin α5β1. (A) IL-1β, (B) IL-8, and (C) TNF-α protein levels in Ca9-22 cells pretreated with anti-integrin α5β1 IgG and then stimulated with *P*. *gingivalis* LPS for 24 h. Differences among groups were analyzed using one-way ANOVA. Data are expressed as means ± SD (*n* = 4). *p < 0.05 vs. control. ND: not detectable.

**Fig 7:**
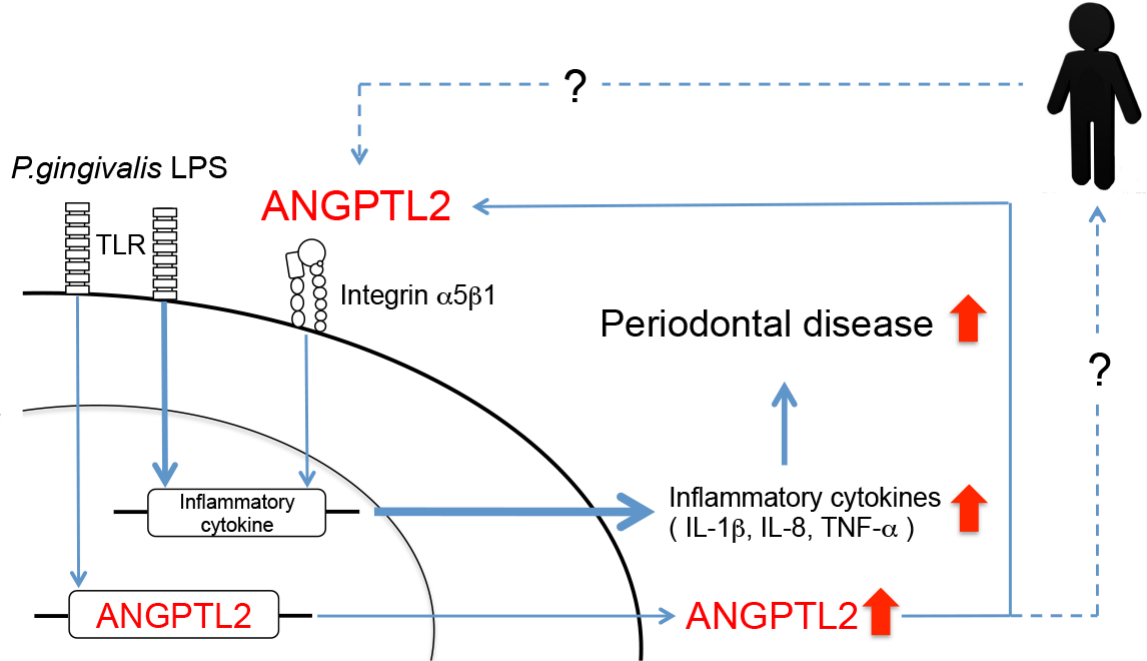
Diagram summarizing the possible mechanisms responsible for ANGPTL2 function in human gingival epithelial cells. We identified a novel signaling cascade comprising sequential *P*. *gingivalis* LPS stimulation → ANGPTL2 induction → integrin α5β1 → inflammatory cytokine induction, which results in potent periodontal disorganization activity in gingival epithelial cells. The bold line is generally considered as the main pathway mediating inflammatory cytokine production by periodontal pathogens such as *P*. *gingivalis* LPS. Non-bold line is shown as a novel signaling pathway via ANGPTL2 in *P*. *gingivalis* LPS stimulation. The dashed line is based on a hypothesis that ANGPTL2 is one of key mediator between periodontal disease and systemic disease.

We next tested whether inflammatory cytokine induction is affected by secreted ANGPTL2 using anti-integrin α5β1 IgG. Pretreatment with anti-integrin α5β1 IgG suppressed IL-1β, IL-8, and TNF-α induction, indicating that an interaction between secreted ANGPTL2 and integrin α5β1 is needed for optimal inflammatory cytokine induction (Fig 6A–6C). Taken together, these data indicate that the *P*. *gingivalis* LPS → ANGPTL2 induction → integrin α5β1 → inflammatory cytokine signaling cascade operates in Ca9-22 cells (Fig 7).

## Discussion

Various substances involved in the progression or activity of periodontal disease, including cytokines (ILs, TNF-α, receptor activator of nuclear factor-κB ligand, and osteoprotegerin) and lipids (prostaglandin E2 and leukotriene), have been detected in GCF [29, 30], and some have been verified as markers of periodontal disease [31, 32]. To date, functional data on the pathophysiological roles of ANGPTL2 in periodontal disease are scarce. In this study, we demonstrated for the first time that ANGPTL2 levels are significantly increased in GCF from CP patients.

ANGPTL2 induction by *Escherichia coli* LPS was previously reported in human retinal pigment epithelial cells [33]. We similarly showed a significant increase in ANGPTL2 mRNA and protein levels after *P*. *gingivalis* LPS stimulation in cultured cells. Since *P*. *gingivalis* is one of the most important periodontal pathogens, it is possible that ANGPTL2 is produced from gingival epithelial cells during periodontitis progression. The presence of an ANGPTL2 signaling cascade in periodontal tissue (and especially in gingival epithelial cells) was previously unknown. TLR proteins are expressed on the surface of periodontal tissue component cells such as gingival epithelial cells and fibroblasts. Previous reports indicate that TLR2 and TLR4 are specifically expressed on periodontal cells [34] and involved in the immune response of periodontal tissues [35]. The unique lipid structure of *P*. *gingivalis* LPS is specifically recognized by both TLR2 and TLR4 [36]. TLR2 and TLR4 knockdown led to significant suppression of ANGPTL2 protein. In addition, TLR2 knockdown had a stronger effect than TLR4 knockdown on ANGPTL2 inhibition, suggesting that TLR2 is the major mediator of *P*. *gingivalis* LPS suppression of ANGPTL2 in Ca9-22 cells.

In vascular endothelial cells or monocytes, ANGPTL2 binding to its receptor, integrin α5β1, induces the production of various inflammatory cytokines. Therefore, increased ANGPTL2 levels can prolong chronic inflammation [16]. We confirmed that integrin α5β1 is constitutively expressed in Ca9-22 cells and observed that pretreatment with anti-integrin α5β1 IgG suppressed inflammatory cytokine induction in epithelial cells, indicating that ANGPTL2-induced overproduction of inflammatory cytokines is mediated by integrin α5β1. ANGPTL2 receptor consists of two forms, integrin α5β1 and LILRB2 [37]. However, we confirmed by flow cytometry that the alternative ANGPTL2 receptor, LILRB2, was not expressed on Ca9-22 cells (S1 Fig). In addition, the expression of LILRB2 was not enhanced upon stimulation with *P*.*gingivalis* LPS or rhANGPTL2. Therefore, we think that the alternative ANGPTL2 receptor, LILRB2, was not involved in the induction of proinflammatory cytokines in Ca9-22 cells.

In rheumatoid arthritis patients, the ANGPTL2 concentration is increased (to about 25 ng/ml) in synovial fluid and the synovium [38]. In addition, Tabata et al. reported that high concentrations (1–20 μg/ml) of rhANGPTL2 did not have a cytotoxic effect on human umbilical vein endothelial or arterial endothelial cells [19]. Thus, we used rhANGPTL2 concentrations in the order of ng/ml to stimulate Ca9-22 cells for clarifying the role of ANGPTL2 in gingival epithelial cells. However, the average ANGPTL2 concentration in GCF was even lower, at about 300 pg/ml (Fig 1). There is a therefore discrepancy between the ANGPTL2 concentration used in *in vitro* experiments and those detected *in vivo*. However, we hypothesize that ANGPTL2 accumulation at sites of inflammation results in high local ANGPTL2 concentrations.

A relationship between periodontal disease and various systemic diseases has been widely reported [7, 8, 10, 12, 13]. For example, it is generally accepted that a two-way relationship exists between periodontal disease and diabetes mellitus, and that TNF-α may mediate this association. The TNF-α level in GCF and serum is an inflammatory marker in CP and type 2 diabetes patients [39]. As ANGPTL2 is constitutively present in the blood and can act systemically [19], we speculate that ANGPTL2 expressed in the periodontium could have systemic effects as well as its role in irreversibly remodeling periodontal tissue. In the present study, ANGPTL2 was also detected in GCF (Fig 1). In addition, ANGPTL2 is constitutively expressed in adipocytes and secreted by lung cancer cells, prostate cancer cells, and peritoneal cells, where it has topical effects [22, 40, 41]. We demonstrated that the ANGPTL2 concentration is higher in GCF from CP patients compared with healthy controls; however, we were unable to investigate a possible correlation between ANGPTL2 concentrations in GCF and serum from patients with CP and/or systemic disease. Therefore, further studies are needed to determine whether ANGPTL2 links CP and systemic diseases.

A schematic diagram illustrating the possible involvement of ANGPTL2 in periodontal disease is shown in Fig 7. In periodontal disease, the bold line is generally considered the main pathway mediating inflammatory cytokine production by periodontal pathogens such as *P*. *gingivalis* LPS. In the current study, we discovered a novel pathway that acts via ANGPTL2. Interestingly, during periodontal disease progression, the pathway has an autocrine effect in which ANGPTL2 acts as an exacerbating factor. ANGPTL2 overexpression might therefore prolong chronic inflammation via an autocrine loop to promote periodontal tissue destruction. The inflammatory cytokine production by *P*. *gingivalis* LPS stimulation was partially suppressed by siRNA or neutralizing antibody against integrin α5β1 in gingival epithelial cells. Although we demonstrated that the key role of ANGPTL2 was inducing inflammatory cytokines upon *P*. *gingivalis* LPS stimulation in gingival epithelial cells, it is likely that ANGPTL2-independent signaling pathways for the production of cytokines exist in the cells. Further studies are therefore necessary to determine how ANGPTL2 functions as a key mediator in periodontal disease and systemic diseases.

## Supporting information

S1 Fig Expression of the alternative ANGPTL2 receptor, LILRB2, on Ca9-22 cells. Ca9-22 cells stimulated with (A) no stimulation, (B) *P*. *gingivalis* LPS or (C) rhANGPTL2 were incubated with LILRB2 Abs (red line) or with the appropriate isotype control (black line) and analyzed by flow cytometry.
(TIF)
