# Supplementary figures and images for: Angiopoietin-like protein 2 regulates Porphyromonas gingivalis lipopolysaccharide-induced inflammatory response in human gingival epithelial cells
Source: PLoS One. 2017 Sep 21;12(9):e0184825. doi: 10.1371/journal.pone.0184825 (PMC5608282; doi:10.1371/journal.pone.0184825)

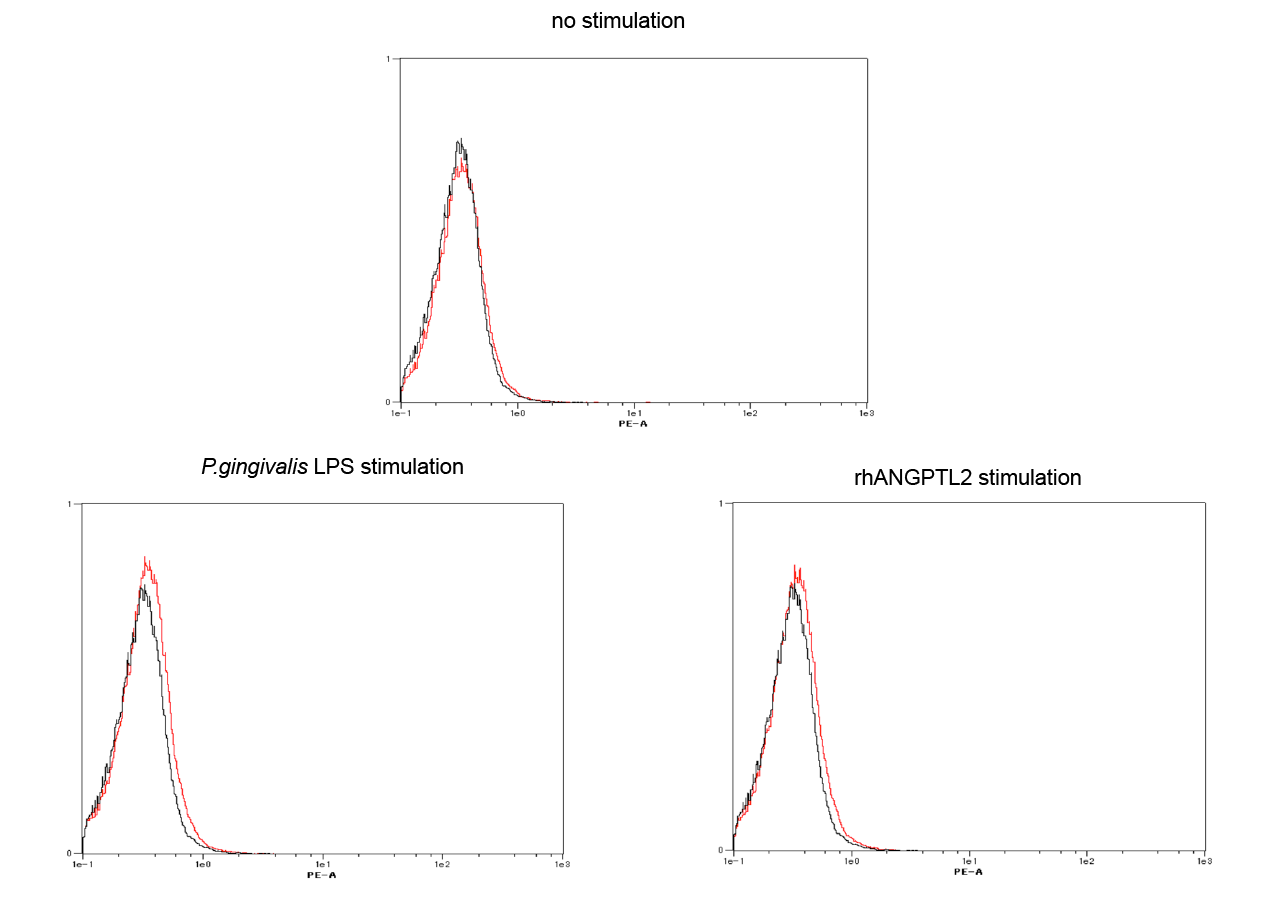

Supplement: S1 Fig — Ca9-22 cells stimulated with (A) no stimulation, (B) P. gingivalis LPS or (C) rhANGPTL2 were incubated with LILRB2 Abs (red line) or with the appropriate isotype control (black line) and analyzed by flow cytometry. (TIF) [file pone.0184825.s001.tif]
